# An Architecture for a Quantum Teleo-Reactive Robot

**DOI:** 10.3390/e28070731

**Published:** 2026-06-27

**Authors:** Antonio Chella, Salvatore Gaglio, Giovanni Pilato, Filippo Vella

**Affiliations:** 1 Dipartimento di Ingegneria (DID), Università degli Studi di Palermo, 90128 Palermo, Italy; antonio.chella@unipa.it (A.C.); salvatore.gaglio@unipa.it (S.G.); 2 Institute of High Performance Computing and Networking (ICAR), Consiglio Nazionale delle Ricerche, 90146 Palermo, Italy

**Keywords:** quantum robots, Hilbert spaces, teleo-reactive robot

## Abstract

A reactive agent operating in a complex environment must classify its perceived state and select an action under uncertainty. This uncertainty may arise from sensor noise, ambiguous perceptual configurations, or the limited separability of the action regions induced by the agent’s policy. We propose a hybrid classical–quantum architecture for a reactive agent in which the perceived state, represented as a classical sensor vector, is mapped onto a quantum feature space. In this space, learned conceptualizations or rule-defined perceptual regions are represented as reference states, and similarities between the current perception and such references are used to support action selection. The architecture is evaluated on a public wall-following robot dataset. Two implementations are considered: (i) a quantum-kernel classifier based on ZZ feature maps and (ii) an illustrative quantum circuit that explicitly encodes sensor conditions into qubits and performs measurement-based action selection. The experimental evaluation is intended as an offline proxy for reactive decision-making, not as a demonstration of a complete closed-loop robotic controller or of quantum advantage. The results show that the proposed framework can represent perceptual ambiguity and connect quantum-state measurement to the selection of discrete reactive actions.

## 1. Introduction

We present a hybrid classical–quantum architecture for reactive action selection in robotic agents. The aim is to investigate how perceptual and proprioceptive information can be mapped into a Hilbert-space representation and then connected to a discrete set of reactive actions.

A robotic platform receives information from heterogeneous sources. Cameras, infrared sensors, lidar, and ultrasonic sensors provide information about the external environment, while internal sensors provide proprioceptive or embodiment-related information, such as battery level, motor currents, and component temperatures. A reactive controller must combine these signals in order to select an action while preserving the operational state of the platform. In the proposed architecture, these heterogeneous inputs are represented as a sensor vector and mapped into a quantum feature space, where similarities among perceptual configurations can be evaluated.

In general, an agent operating in a complex environment may face ambiguous perceptual configurations. A sensed state may be close to more than one learned configuration, and when an option must be chosen, the selection is affected by a certain degree of uncertainty. A quantum system can consider all the configurations at the same time, exploiting its intrinsic parallelism when choosing an action to carry out.

The proposed architecture is inspired by Hilbert-space models of cognition and decision-making, including the framework discussed by Faggin and D’Ariano [1]. In the present work, however, we do not address philosophical claims about consciousness or free will. We use the quantum formalism operationally, as a representational and computational framework for mapping perceptual states onto a Hilbert space and selecting actions through similarity evaluation or measurement.

The basis or reference states of this space represent learned conceptualizations or rule-defined perceptual regions. The perceived state is mapped into the same space, and its similarity to the reference states is used to determine the corresponding action. When several action alternatives have non-negligible activation, the measurement process provides a stochastic mechanism for selecting a definite classical command from the quantum representation.

The Nilsson formalism for teleo-reactive (T-R) robots provides a compact way to specify goal-directed behavior through prioritized condition–action rules [2]. A T-R program continuously re-evaluates the conditions associated with its rules and executes the action corresponding to the highest-priority rule whose condition is satisfied [2,3]. In this work, the quantum component is not intended to replace the whole T-R control loop. Rather, it is used to implement the perceptual evaluation and action-selection layer, where conditions may be represented as regions or reference states in a Hilbert space.

Other works in the literature have also proposed architectures that combine classical and quantum computation, leveraging the strengths of both approaches [4,5].

In Section 2, the reactive agents are presented and the high-dimensional spaces are described. In Section 3, the proposed architecture is presented along with the rationale of our approach. The employed data and the experimental results are shown in Section 4 and Section 5. The conclusions are drawn in Section 6.

## 2. Background

This section provides theoretical background on classical reactive agents and on the use of kernels in cognitive systems.

### 2.1. Classical Reactive Agents

Reactive agents [6,7,8] are based on a direct sense–act principle: the agent perceives a condition in the surrounding environment and immediately executes the associated action, without relying on deliberation, internal planning, or explicit world models. This makes them substantially different from deliberative agents, where action selection is driven by internal representations and by the evaluation of alternative courses of action.

These systems are usually organized as sets of concurrent condition–action rules, which are activated when specific environmental signals are detected. The environment itself plays the role of operational guide: the agent continuously senses and reacts to local changes, without maintaining an internal representation of the world and without exploiting past experience. As a consequence, reactive agents are particularly fast, since they avoid the computational overhead associated with reasoning, prediction, and comparison among alternative plans. This feature makes them suitable for dynamic and unpredictable settings, as well as for repetitive tasks where timely response is more relevant than long-term planning.

The behavior of a reactive agent emerges from its situated and embodied interaction with the environment. Its actions are therefore strongly dependent on the immediate context, rather than on abstract symbolic processing. The most influential realization of this paradigm is the subsumption architecture introduced by Rodney Brooks [9], where the agent behavior is structured into parallel layers, each devoted to a specific capability, such as obstacle avoidance or navigation. These layers coordinate by suppressing or overriding one another, so that higher layers can subsume lower-level outputs in order to obtain more complex behaviors while preserving fast reactive responses at the basis.

However, reactive agents are intrinsically limited by the absence of explicit goals, memory, and foresight. They respond only to the current perceptual situation, and this prevents them from effectively addressing tasks requiring planning, strategy, or learning from experience. Without a goal-oriented direction and without memory of previous states, the agent may repeat the same actions, remain trapped in local behavioral loops, or fail to progress in long-horizon tasks. Thus, the same simplicity that ensures efficiency in immediate response becomes a limitation when complex problem solving requires persistence, anticipation, and sustained goal-directed behavior.

### 2.2. Kernels and High-Dimensional Linear Spaces

Let us consider a cognitive system that receives a stream of perceptual inputs for which no fixed symbolic description is available. Some percepts may exhibit higher similarity than others, although no explicit rule is given in advance for determining their relations. In this setting, the system relies on a collection of previously encountered perceptual instances, together with the capability of assessing degrees of similarity among them, instead of exploiting predefined defining criteria.

From this perspective, perception is governed by the relational structure induced among perceptual states, instead of being organized through rule-based categorization. The central problem is therefore represented by the construction of a suitable representational space in which perceptual states can be meaningfully compared, combined, and transformed.

Formally, let (D) denote the domain of perceptual inputs. The task is to define a similarity structure over (D) that captures the relevant perceptual relations without requiring an explicit feature decomposition. This can be achieved by introducing a kernel function

(1) k:D×D→R,

which assigns to each pair of percepts a real-valued measure of similarity [10].

The kernel provides a computational mechanism for encoding perceptual similarity, capturing relational patterns without requiring explicit features. Through the kernel approach, this similarity function implicitly defines a mapping

(2) σ:D→H

into a Hilbert space (H), where the inner product corresponds to similarity:

(3) $$k\left(a,b\right)={\langle \sigma \left(a\right),\sigma \left(b\right)\rangle }_{H}.$$

The mapping itself need not be computed explicitly; the kernel alone is sufficient to determine the geometry of perceptual states.

In a quantum implementation, a perceptual input can be encoded as a quantum state within a Hilbert space, by exploiting one or more qubits according to the dimensionality of the input and to the adopted feature map. The resulting state may contain superposed components associated with different regions, meanings, or interpretations of the perceptual space. This representation allows ambiguity and graded similarity to be preserved, since multiple potential interpretations can coexist before the selection of the final action.

Similarity between percepts is geometrically captured by inner products between qubit vectors. If ($w_{a}=\sigma \left(a\right)$) and ($w_{b}=\sigma \left(b\right)$), their normalized forms

(4) $${\bar{w}}_{a}=\frac{w_{a}}{|w_{a}|},\;{\bar{w}}_{b}=\frac{w_{b}}{|w_{b}|}$$

satisfy

(5) $$\langle {\bar{w}}_{a},{\bar{w}}_{b}\rangle =\cos\theta ,$$

where (θ) is the angle between the vectors. Smaller angles indicate greater similarity, and larger angles greater dissimilarity, grounding perceptual relations in the geometry of the Hilbert space.

This framework enables quantum-like operations over perceptual states. In particular, superposition mechanisms allow interference effects and contextual dependencies to emerge as intrinsic properties of the perceptual representation.

Only at the point of measurement or environmental interaction does a projection occur, selecting a specific perceptual configuration. Until then, the system maintains a rich representational structure in which multiple interpretations coexist, fully compatible with quantum-computational manipulation of perceptual information.

In the papers [11,12], the adoption of Grover’s algorithm makes it possible to find the best path that a robot can take to reach a target position. The selected algorithm, employing quantum techniques, can solve a search in a space with *N* elements in $\sqrt{N}$ steps instead of O(N), which is the classical complexity. Grover’s algorithm uses superposition and entanglement to represent all the possible candidates; the application of an “oracle” (a function checking for the solution) and a diffusion operator amplifies the correct solutions and discards all the others.

Section 3 introduces the proposed hybrid architecture and describes how the quantum component is connected to teleo-reactive action selection.

## 3. Proposed Hybrid Quantum-Classical Architecture: The Q-Reactive Agent

The proposed architecture relies on the information that a robot can acquire from both external and internal sources. A robot situated in the physical world receives heterogeneous perceptual inputs: temperature sensors provide scalar values, sonar and lidar sensors indicate the presence and distance of obstacles, and cameras provide multidimensional visual data.

These data can be represented as a sensor vector that is continuously updated according to the readings acquired at each time step. In addition to information about the external environment, the robot may also acquire proprioceptive and embodiment-related signals, such as motor currents, battery level, and component temperatures. Together, these values define the state of the robot at a given time *t*.

The perceptual and proprioceptive signals acquired by the robot are initially classical numerical values. In the proposed architecture, these values are collected into a sensor vector and then mapped onto a Hilbert space by means of a quantum feature map or a dedicated quantum circuit. Since the input channels convey a stream of data, an information mapping is needed to manage this large amount of information and make the information processing manageable with reduced resources. We consider possible to achieve this mapping with a quantum kernel to pinpoint centroids identifying relevant input patterns that may recur during the robot’s normal operations.

**Figure 1:**
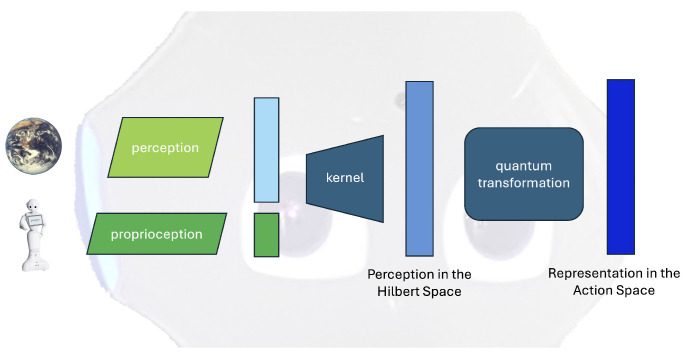
Architecture for quantum processing of perception and proprioception and mapping onto the action space.

The application of a quantum kernel is useful for multiple reasons. Kernels provide a mechanism for comparing and clustering input patterns [13], and they can be applied to heterogeneous inputs coming from both external sensors and internal proprioceptive signals. The use of quantum gates introduces correlations between input dimensions, which can capture dependencies between internal and external signals without implying a strict inseparability. This processing can be carried out with an architecture such as the one depicted in Figure 1. The values from the perception process are mapped onto the Hilbert space through a kernel. For the quantum-kernel implementation considered here, the feature map is fixed and is not trained. The second implementation, described below, instead uses a dedicated circuit for rule-based action selection. To clarify the relationship between perception, conceptualization and action selection, we formalize the mapping as follows.

### Formal Mapping from Perception to Action

Let $x\in R^{d}$ denote the input vector of sensor readings. The input is mapped into a Hilbert space through a fixed quantum feature map, producing a state |ϕ(x)〉. A set of reference states $\left\{|c_{i}\rangle \right\}$ represents learned conceptualizations.

Similarity scores are computed as

(6) $$s_{i}=\left|\langle \phi \left(x\right),c_{i}\rangle \right|$$

**Figure 2:**
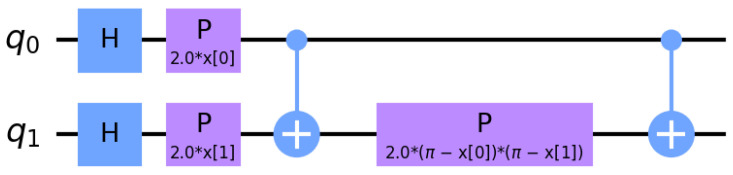
Example of a ZZ feature map with linear entanglement for data with dimension 2.

An implementation of the mapping of the sensor values in the Hilbert space is carried out through ZZ feature maps. Figure 2 shows the ZZ Feature Map for bidimensional data. The same kernels, following the data dimension, can be replicated downstream, increasing the expressiveness of the quantum feature space [14].

The representation in the Hilbert space can be linked to a set of actions that the robot can execute. According to the value measured by the sensors, the robot can perform a given action, and when the input changes values, a different action can be triggered. The representation in the action space allows to consider a quantum representation for the possible actions of the robot.

**Figure 3:**
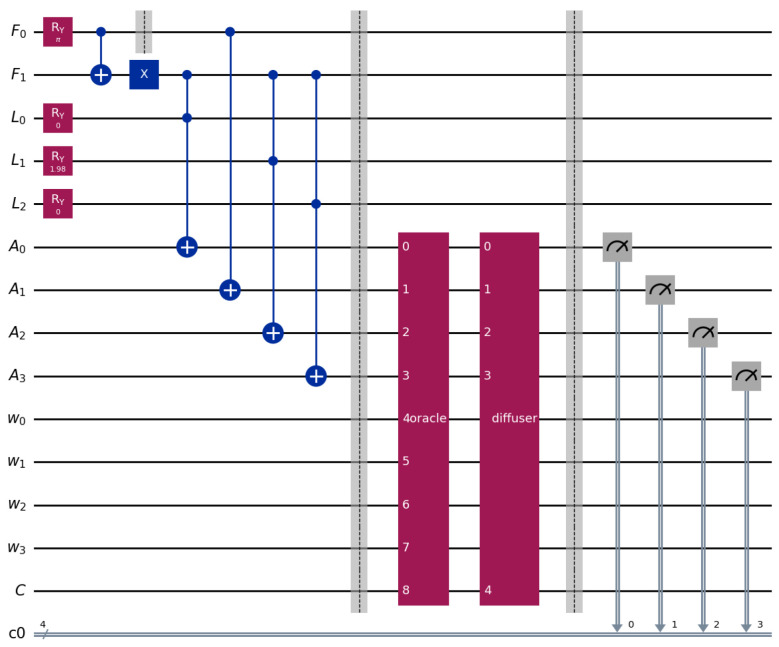
Illustrative quantum circuit for rule-based action selection from front and left distance sensors. Sensor conditions are encoded into qubits, propagated to action qubits, amplified through an oracle–diffuser block, and finally measured to obtain a classical action.

In this formulation, either learned conceptualizations or rule-defined perceptual regions can be represented as reference states in the Hilbert space. Each reference state is associated with a reactive action. The action is selected through a measurement-like projection in the action space. In this way, the uncertainty in the feature space is maintained up to the action selection when the action is chosen, according to the probability distribution induced by superposition. An illustrative circuit realization of this second possibility is shown in Figure 3.

In this work, we explore two concrete implementations of this mapping:

1. A quantum-kernel method using ZZ feature maps, where the perceptual state is mapped onto a quantum feature space and the action label is inferred by a classical support vector classifier;
2. An illustrative quantum circuit that encodes sensor readings into qubits, propagates condition activations to action qubits, applies an oracle–diffuser block to amplify valid single-action configurations, and performs an explicit measurement to select the action.

The first implementation is suitable for learning from labeled data. The second implementation is not intended to demonstrate quantum advantage but to make explicit how a measurement-based action-selection mechanism can be embedded in the proposed architecture. Both are evaluated in Section 5.

## 4. The Dataset

We have used a publicly available dataset named “Sensor readings from a wall-following robot” [15,16]. The dataset was generated during a controlled mobile robotics experiment designed to investigate the complexity of a navigation task. The main idea behind this experiment was that the wall-following task, despite seeming simple, is actually a non-linearly separable classification problem.

In particular, data were recorded as a SCITOS G5 robot autonomously navigated an indoor environment by executing a clockwise wall-following behavior for four complete laps around a room.

The robot was equipped with 24 ultrasonic sensors arranged radially around its chassis. During the experiment, raw distance measurements from all 24 sensors were sampled at a frequency of 9 Hz. At the same time, a human operator annotated each time step with the corresponding navigation command being executed by the robot (e.g., moving forward, turning left, or turning right).

In order to support a comparative analysis of classifier performance with respect to input dimensionality, the acquired data were processed into three distinct datasets, each characterized by the same number of temporal samples.

The first dataset contains the raw, unprocessed readings collected from all ultrasonic sensors, and is used as a baseline for evaluating classification in a high-dimensional input space. The second dataset is obtained by grouping the ultrasonic sensors into four directional categories. More precisely, the space surrounding the robot is partitioned into four 60-degree arcs, oriented toward the front, left, right, and rear directions. This representation provides four simplified features for each time step, namely front distance, left distance, right distance, and rear distance. Finally, the third dataset retains only two of these simplified readings, i.e., front distance and left distance, with the aim of evaluating classifier performance under limited input dimensionality conditions.

**Figure 4:**
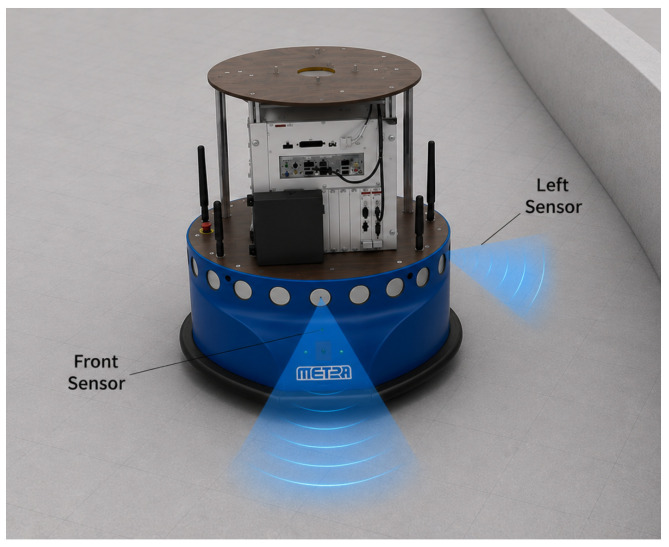
SCITOS G5 robot and positions of the front and left sensors (image created with ChatGPT 5.5).

All three datasets were recorded simultaneously; consequently, each file contains the same number of rows, with each row corresponding to a specific sampling time step and its associated class label. In Figure 4 is depicted the robot SCITOS G5 with a visualization of the front and left sensors.

## 5. Experimental Evaluation

### 5.1. Quantum Kernel Classification

The experimental evaluation focuses on the classification of sensor states, which is adopted as a proxy task for action selection within the proposed architecture. This simplified setting makes it possible to isolate the representational role of the quantum feature map without implementing a full closed-loop robotic control system. Consider this simple robot as an illustrative example. The main task of the robot is to follow the wall of its environment, exploiting the information provided by its sensors [16].

In this formulation, we do not consider the details of the wall-following task, considering that the implementation can be accomplished by using simple robotic platforms and processing the sensor information with classical models.

All experiments were conducted offline on the labeled wall-following dataset. The input features were normalized before kernel computation. For the quantum-kernel experiments, we considered the two-sensor and four-sensor versions of the dataset. The quantum kernel matrix was computed from the ZZ feature map and then used by a Support Vector Classifier. A classical SVC with a radial basis function (RBF) kernel was used as a baseline. The same train–test partition was used for the compared methods. The reported metrics are accuracy, macro precision, macro recall, macro F1, and weighted F1. No claim of quantum advantage is made from these experiments; the goal is to assess whether the proposed representation can support action-label prediction and measurement-based action selection in a simplified offline setting.

An important aspect of the task is selecting the robot’s policy, which is processed using quantum methods. During operation, the robot may operate multiple modalities according to the sensor inputs, which can provide both external and internal information.

In the simplest formulation of the wall-following task, only two sensor readings are considered: the front distance *F* and the left distance *L*.

In this case, the following set of rules can be selected to form a simple process for determining the actions to choose.

(7) $$\begin{array}{ccccc} F\leq \theta_{f}\; & & & \to & \mathrm{Sharp}\text{-}\mathrm{Right}\text{-}\mathrm{Turn},\\ F>\theta_{f} & \wedge & L>\theta_{l}^{\mathrm{high}} & \to & \mathrm{Slight}\text{-}\mathrm{Left}\text{-}\mathrm{Turn},\\ F>\theta_{f} & \wedge & \theta_{l}^{\mathrm{low}}<L\leq \theta_{l}^{\mathrm{high}} & \to & \mathrm{Move}\text{-}\mathrm{Forward},\\ F>\theta_{f} & \wedge & L\leq \theta_{l}^{\mathrm{low}} & \to & \mathrm{Slight}\text{-}\mathrm{Right}\text{-}\mathrm{Turn}. \end{array}$$

**Figure 5:**
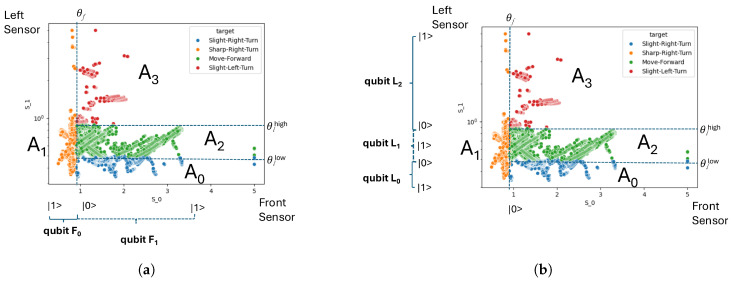
Rule-induced partition of the two-sensor perceptual space and corresponding qubit encoding. The thresholds $\theta_{f}$, $\theta_{l}^{\mathrm{low}}$, and $\theta_{l}^{\mathrm{high}}$ define perceptual regions associated with the actions $A_{0}$ (Slight-Right-Turn), $A_{1}$ (Sharp-Right-Turn), $A_{2}$ (Move-Forward), and $A_{3}$ (Slight-Left-Turn). (**a**) Front-sensor thresholding and encoding into $F_{0}$ and $F_{1}$. (**b**) Left-sensor thresholding and encoding into $L_{0}$, $L_{1}$, and $L_{2}$.

Figure 5 illustrates the rule-induced partition of the two-sensor perceptual space and the corresponding qubit encoding. The front distance *F* is associated with two condition qubits, $F_{0}$ and $F_{1}$, representing, respectively, the case in which the robot is too close to a frontal obstacle and the case in which the forward direction is sufficiently clear. The left distance *L* is associated with three condition qubits, $L_{0}$, $L_{1}$, and $L_{2}$, corresponding, respectively, to the robot being too close to the left wall, at an appropriate wall-following distance, and too far from the wall. This visualization clarifies how the symbolic conditions in Equation (7) are translated into qubit activations before the action-selection circuit is applied.

**Figure 6:**
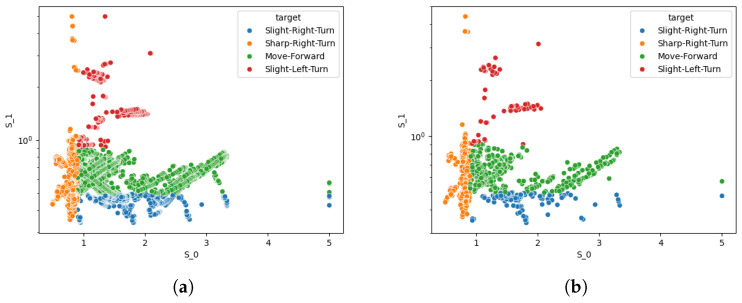
Plots of a portion of the dataset. Training set (**a**) and test set (**b**).

In Figure 6, the plots of a portion of the dataset with two sensors is shown. The x axis corresponds to the *front* sensor and the y axis to the *left* sensor. The left plot represents the values from the training set, and the right plot represents the values for the test set is shown. The colors correspond to the actions of the robot.

The values obtained with the ZZ feature map are shown in Table 1. The results were obtained in the case with two sensors and the case with four sensors. The ZZ feature map was employed either in simple form or with a replication. In this latter case, the first set of quantum gates was replicated with an identical set of gates next to the first set. The ability to assign the correct label according to the read values is reported in the table. It appears that the case with two sensors is easier than the case with four sensors, and the metric results, namely, accuracy, precision, recall, and F1, are higher for the case of two sensors than four sensors. Furthermore, the repetition of the ZZ feature map appears not to be beneficial. The metrics of accuracy, precision, recall and F1 with a single ZZ feature map are always higher than the values with a repetition, and so the single application of the kernel is chosen.

The RBF baseline provides a classical comparison for the two-sensor case. The single ZZ feature map obtains higher scores than the RBF baseline in this setting, whereas the replicated ZZ feature map does not improve performance. This suggests that increasing circuit depth through a simple repetition of the feature map is not beneficial for this dataset.

These results should be interpreted cautiously. The purpose of the comparison is not to claim quantum advantage but to evaluate whether the proposed quantum feature representation can support the classification of perceptual states associated with reactive actions. The kernel-based method, however, still relies on a classical SVC for the final label assignment and does not explicitly implement a quantum measurement for action selection. For this reason, we next introduce a second implementation based on an explicit quantum circuit and measurement of action qubits.

### 5.2. Quantum Circuit with Explicit Measurement for Action Selection

In this subsection, we present a quantum circuit that implements the mapping from sensor readings to actions through explicit quantum state preparation, a Grover-inspired oracle–diffuser block, and measurement.

#### 5.2.1. Action Rules and Thresholds

We adopt the same rule set defined in Equation (7). In the circuit implementation, the four reactive actions are associated with four action qubits according to the following mapping:

(8) $$\begin{array}{ccc} A_{0} & = & \mathrm{Slight}\text{-}\mathrm{Right}\text{-}\mathrm{Turn},\\ A_{1} & = & \mathrm{Sharp}\text{-}\mathrm{Right}\text{-}\mathrm{Turn},\\ A_{2} & = & \mathrm{Move}\text{-}\mathrm{Forward},\\ A_{3} & = & \mathrm{Slight}\text{-}\mathrm{Left}\text{-}\mathrm{Turn}. \end{array}$$

This ordering is used consistently in the circuit and in the confusion matrix.

#### 5.2.2. Encoding of Sensor Values into Qubits

The front sensor value is encoded into two qubits, $F_{0}$ and $F_{1}$. Qubit $F_{0}$ is rotated towards |1〉 when the front distance is below $\theta_{f}$ (robot too close), using an $R_{y}$ gate with an angle proportional to the sensor reading. Qubit $F_{1}$ represents the opposite condition (front distance safe) and is obtained via a CNOT gate and an X gate.

The left sensor is encoded into three qubits, $L_{0}$, $L_{1}$, and $L_{2}$:

- $L_{0}$ is close to |1〉 when $L\leq \theta_{l}^{\mathrm{low}}$, corresponding to the robot being too close to the wall;
- $L_{1}$ is close to |1〉 when $\theta_{l}^{\mathrm{low}}<L\leq \theta_{l}^{\mathrm{high}}$, corresponding to the desired wall-following distance;
- $L_{2}$ is close to |1〉 when $L>\theta_{l}^{\mathrm{high}}$, corresponding to the robot being too far from the wall.

Each qubit is prepared by an $R_{y}$ gate whose rotation angle is a function of the sensor reading, so that the probability of measuring |1〉 increases as the sensor value moves deeper into the corresponding region.

The correspondence between the sensor thresholds, the perceptual regions, and the condition qubits is illustrated in Figure 5.

#### 5.2.3. Action Qubits and Oracle–Diffuser Block

Four action qubits, $A_{0},\ldots ,A_{3}$, represent the candidate actions in the order defined in Equation (8). Controlled and multi-controlled gates propagate the activation of the sensor-condition qubits to the corresponding action qubits. In particular, $A_{1}$ is activated when $F_{0}$ indicates that the robot is too close to an obstacle in front; $A_{0}$ is activated when the front direction is safe and the robot is too close to the left wall; $A_{2}$ is activated when the front direction is safe and the left distance lies in the desired interval; and $A_{3}$ is activated when the front direction is safe and the robot is too far from the left wall.

After the condition-to-action propagation, an oracle–diffuser block inspired by Grover’s search algorithm is applied to the action register. The oracle marks configurations in which exactly one action qubit is active. The diffuser then increases the probability of measuring a valid single-action configuration. This block does not guarantee that a single action will always be measured, but it biases the measurement toward valid action configurations.

Finally, a measurement is performed on the action qubits. If the measurement yields a state with exactly one active action qubit, the corresponding action is selected. If the measurement yields an invalid configuration, such as more than one active action qubit or all action qubits equal to zero, a default action is executed. In the present experiments, the default action is Move-Forward. Figure 3 shows the full circuit.

#### 5.2.4. Confusion Matrix

We evaluated the circuit on the test set of the two-sensor dataset. The resulting confusion matrix is shown in Table 2. The circuit selects the correct action in most cases. The remaining errors occur close to the threshold boundaries between adjacent action regions, where small changes in the encoded sensor values can affect the measured action.

The circuit obtains an accuracy of approximately 0.912 on the two-sensor test set and a weighted F1 of 0.904. This value should not be directly compared with the kernel-based SVC results as evidence of superiority, because the circuit implements a hand-defined rule partition of the perceptual space, whereas the SVC models are learned from labeled data. The purpose of this implementation is therefore to illustrate a complete quantum-compatible action-selection process based on encoding, oracle-based amplification, and final measurement, more than to achieve state-of-the-art performance or to demonstrate quantum advantage.

This simplified setup does not implement a full robotic control loop, a task that is left for future work.

## 6. Conclusions

We have presented a hybrid classical–quantum architecture for reactive action selection. In the proposed framework, perceptual states are mapped onto a Hilbert space, where they can be compared with learned conceptualizations or rule-defined perceptual regions associated with reactive actions.

Two implementations were explored on a public wall-following robot dataset. The first uses a ZZ feature map quantum kernel combined with a Support Vector Classifier. This experiment evaluates the classification of sensor states as an offline proxy for reactive action selection. The second implementation uses a dedicated quantum circuit in which front and left sensor values are encoded into qubits, condition activations are propagated to action qubits, an oracle–diffuser block amplifies valid single-action configurations, and a final measurement selects a classical action.

The results show that the proposed architecture can support both data-driven classification through quantum kernels and explicit measurement-based action selection through a quantum circuit. The comparison with a classical RBF-kernel SVC is included as a baseline, but no claim of quantum advantage is made. The present evaluation does not implement a full closed-loop robotic controller; rather, it provides an offline validation of the representational and action-selection components of the architecture.

Future work will focus on integrating the proposed module into a closed-loop robotic platform or simulator, extending the approach to richer perceptual and proprioceptive inputs, and investigating learnable quantum circuits that combine the flexibility of data-driven methods with explicit measurement-based action selection.

## Figures and Tables

**Table 1:** Classification results for data from two and four sensors using a Support Vector Classifier with different kernels.

Sensors	Kernel	Accuracy	Precision	Recall	F1	Weighted F1
2	RBF (classic)	0.849	0.654	0.612	0.619	0.814
2	single ZZ	0.895	0.776	0.776	0.776	0.898
2	double ZZ	0.774	0.774	0.774	0.774	0.837
4	single ZZ	0.631	0.631	0.631	0.631	0.672
4	double ZZ	0.607	0.607	0.606	0.607	0.624

**Table 2:** Confusion matrix for the quantum circuit with explicit measurement. Rows correspond to true actions, and columns correspond to selected actions.

	Slight-Right	Sharp-Right	Move-Forward	Slight-Left
Slight-Right	449	0	0	0
Sharp-Right	0	418	8	0
Move-Forward	13	0	49	0
Slight-Left	76	0	0	87

## Data Availability

Quantum kernel projections of the wall following robot are available, through the Zenodo platform, at the link [17].
